# Delayed cold-stored vs. room temperature stored platelet transfusions in bleeding adult cardiac surgery patients—a randomized multicentre pilot study (PLTS-1)

**DOI:** 10.1186/s40814-024-01518-z

**Published:** 2024-06-15

**Authors:** Justyna Bartoszko, Miki Peer, Deep Grewal, Saba Ansari, Jeannie Callum, Keyvan Karkouti

**Affiliations:** 1 Department of Anesthesia and Pain Management, Sinai Health System, Women’s College Hospital, University Health Network, Toronto, ON Canada; 2 Department of Anesthesiology and Pain Medicine, University of Toronto, Toronto, ON Canada; 3 Peter Munk Cardiac Centre, University Health Network, Toronto, ON Canada; 4 University of Toronto Quality in Utilization, Education and Safety in Transfusion Research Program, Toronto, ON Canada; 5 Department of Pathology and Molecular Medicine, Kingston Health Sciences Centre and Queen’s University, Kingston, ON Canada; 6 Interdepartmental Division of Critical Care, Department of Medicine, University of Toronto, Toronto, ON Canada

**Keywords:** Cold-stored platelets, Cardiac surgery, Transfusion, Hemostasis, Platelets, Thrombocytes

## Abstract

**Background:**

Platelets stored at 1–6 °C are hypothesized to be more hemostatically active than standard room temperature platelets (RTP) stored at 20–24 °C. Recent studies suggest converting RTP to cold-stored platelets (Delayed Cold-Stored Platelets, DCSP) may be an important way of extending platelet lifespan and increasing platelet supply while also activating and priming platelets for the treatment of acute bleeding. However, there is little clinical trial data supporting the efficacy and safety of DCSP compared to standard RTP.

**Methods:**

This protocol details the design of a multicentre, two-arm, parallel-group, randomized, active-control, blinded, internal pilot trial to be conducted at two cardiac surgery centers in Canada. The study will randomize 50 adult (≥ 18 years old) patients undergoing at least moderately complex cardiac surgery with cardiopulmonary bypass and requiring platelet transfusion to receive either RTP as per standard of care (control group) or DCSP (intervention group). Patients randomized to the intervention group will receive ABO-identical, buffy-coat, pathogen-reduced, platelets in platelet additive solution maintained at 22 °C for up to 4 days then placed at 4 °C for a minimum of 24 h, with expiration at 14 days after collection. The duration of the intervention is from the termination of cardiopulmonary bypass to 24 h after, with a maximum of two doses of DCSP. Thereafter, all patients will receive RTP. The aim of this pilot is to assess the feasibility of a future RCT comparing the hemostatic effectiveness of DCSP to RTP (defined as the total number of allogeneic blood products transfused within 24 h after CPB) as well as safety. Specifically, the feasibility objectives of this pilot study are to determine (1) recruitment of ≥ 15% eligible patients per center per month); (2) appropriate platelet product available for ≥ 90% of patients randomized to the cold-stored platelet group; (3) Adherence to randomization assignment (> 90% of patients administered assigned product).

**Discussion:**

DCSP represents a promising logistical solution to address platelet supply shortages and a potentially more efficacious option for the management of active bleeding. No prospective clinical studies on this topic have been conducted. This proposed internal pilot study will assess the feasibility of a larger definitive study.

**Trial registration:**

NCT 06147531 (clinicaltrials.gov).

## Background

Platelets are one of the most commonly used blood products and as such are frequently in short supply. Currently, platelets are stored at room temperature (20–24 °C) to retain post-transfusion circulatory longevity but at the cost of reduced shelf-life, which is part of the reason for the supply challenges. Cold storage (at 1–6 °C) of platelets prolongs their shelf-life while maintaining hemostatic effectiveness in acutely bleeding patients. Thus, converting room temperature-stored platelets (RTSP) before their shelf-life expires to cold-stored platelets may be a good strategy for extending their shelf-life and addressing the platelet supply challenges, but the safety and comparative effectiveness of this strategy in bleeding patients has not been robustly assessed. This pilot study will assess the feasibility of conducting a definitive comparative study of standard room temperature-stored platelets (RTSP) and delayed cold-stored platelets (DCSP).

Platelet transfusion is the primary therapy for platelet-related bleeding (i.e., excessive bleeding due to platelet deficiency or dysfunction). Platelets are one of the most commonly used blood products, particularly in major cardiothoracic surgery with cardiopulmonary bypass where platelet deficiency and dysfunction occur in all patients to varying degrees [1, 2]. As a result, approximately one in four cardiac surgical patients receive at least one platelet transfusion to treat active bleeding in the operating room or soon thereafter, with high levels of variability across institutions. Approximately 10% of the total platelet supply is directed to cardiac surgery alone [3–5]. Since the 1970s, standard international practice has been to store platelet units at room temperature (20–24 °C). This preserves the longevity of platelets post-transfusion but limits their storage shelf-life to 7 days for both pooled buffy-coat bacterially cultured and pooled buffy-coat pathogen-inactivated platelets [6]. The short shelf-life of room-temperature platelets (RTP) creates major supply challenges for blood suppliers and hospitals and leads to wastage of up to 30% of platelet units due to outdating [7]. Additionally, existing data suggests that storage at room temperature, while prolonging circulatory survival after transfusion, may in fact impair hemostatic function [8].

Alternatively, platelets may be stored at 1–6 °C, referred to as cold-stored platelets (CSP). It has long been known that cold storage of platelets extends their shelf-life (potentially up to 21 days) and reduces the risk of bacterial contamination while maintaining platelet activity and function. Of interest, when platelets are chilled, their metabolic rate is decreased and metabolic substrates (e.g., glucose, lactate) required for energetically demanding hemostatic functions are better preserved [9]. The cumulative structural, metabolic, and molecular changes observed suggest that cold-stored platelets are ‘primed’ for hemostatic activity, and may therefore trigger more rapid formation of lysis-resistant clots than platelets stored at room temperature [8, 10–17]. This altogether suggests that CSP may be superior to conventional room temperature platelets for the management of acute surgical bleeding.

Given that diverting even a small fraction of freshly collected platelets to cold storage would further limit an already strained platelet supply, scientists at Canadian Blood Services (CBS) and elsewhere [18] have investigated refrigerating standard RTP several days after collection to produce delayed cold-stored platelets (DCSP) [9, 19]. Several investigators have shown that DCSP retains an acceptable metabolic and hemostatic profile compared with platelets cold-stored immediately after collection [7, 9, 20]. Overall, shifting platelets from room temperature to cold storage by the end of the 4th day appears to represent an ideal balance between quality preservation and supply maximization [20] that warrants clinical investigation.

Our central hypothesis is that compared to conventional room-temperature platelets, delayed cold-stored platelets have a non-inferior safety profile (due to inhibition of bacterial growth at colder temperatures) and have non-inferior hemostatic efficacy in acutely bleeding surgical patients. In line with this hypothesis, we anticipate that cold-stored platelets will be logistically superior for blood banks as they can be placed in the fridge and do not require space on the agitator, thereby allowing for easier maintenance of ABO-identical stock. The proposed pilot study is designed to determine optimal trial procedures, including the provision and distribution of cold-stored platelets at participating hospitals, for a planned future definitive trial with the hypothesis/hypotheses outlined above. The *primary feasibility outcomes* are (1) adequate recruitment (defined as ≥ 15% of eligible patients recruited per centre per month); (2) adequate cold-stored platelet supply (defined as an appropriate product available at the time of surgery for ≥ 90% of patients randomized to the cold-stored platelet group); (3) adequate clinician adherence to randomization assignment (defined as > 90% of all patients are administered the assigned product).

## Methods

### Design

This protocol is based on reporting guidelines from the SPIRIT guideline for Standard Protocol Items for Clinical Trials [21], adapted as recommended when reporting protocols of feasibility trials [22].

**Fig. 1:**
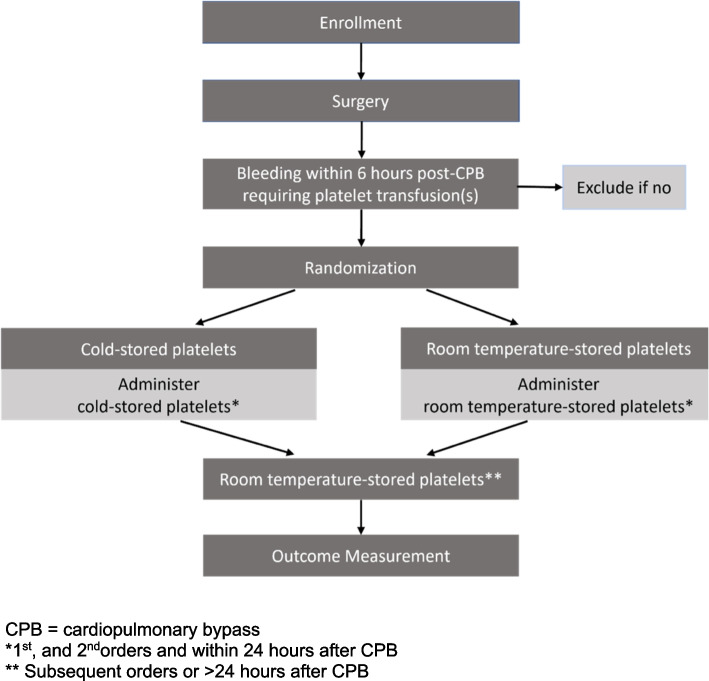
Study flow

PLTS-1 is a multicentre, randomized, active-control, blinded, internal pilot trial, using a conventional, parallel-group, two-armed design to be conducted at two study centers. The study will assess the feasibility of a possible future, definitive RCT designed to determine whether DCSPs are non-inferior to conventional RTP with respect to hemostatic effectiveness (total number of allogeneic blood products transfused within 24 h after CPB) and safety. The study will enroll approximately 150 patients to treat 50 adult (≥ 18 years old) patients undergoing at least moderately complex cardiac surgery with CPB and requiring platelet transfusion. Based on historical institutional data, approximately 30% of patients undergoing complex surgery will require platelet transfusion [23]. We will explore the feasibility of the definitive RCT protocol with respect to participant recruitment, the ability of hospital blood banks to supply ABO-identical DCSP and clinician adherence to randomization assignment. If we proceed to the larger trial with no protocol changes or with protocol changes that can be reasonably expected to not impact the primary endpoints of the definitive trial (as determined by the study steering committee with recommendations from the IDSMC taken into account), the relevant outcome data from the pilot study will contribute to the definitive trial’s dataset thereby establishing this as an internal pilot. A diagram of the proposed study and the outcome assessment is provided in Fig. 1 and Table 1.

**Table 1:** Flow chart of assessments performed throughout the study

Procedures	Before surgery	Surgery	Each platelet administration	POD-1	POD-7 or DC	POD-30
Obtain consent	X					
Inclusion and exclusion criteria	X					
Randomization criteria			x^b^			
Treatment criteria			x			
Platelets stored	X					
Intervention administered			x			
Baseline data						
Demographics	X					
Medical history	X					
Concomitant medications	X					
Surgical data						
Procedure details, date	X					
Intraoperative medications		x				
CPB time		x				
Transfusion data						
Platelets			x	x	x	x
Red cells				x	x	x
Plasma				x	x	x
Hemostatic agents				x	x	
Laboratory assessments						
Complete blood count	X	x	x	x	x	X
Platelet count (total, functioning)	X	x	x			
Coagulation profile ^a^		x	x			
Chemistry	X	x	x	x	x	X
Hematology	X	x	x	x	x	X
Safety labs	X	x	x	x	x	x
Bleeding						
Bleeding severity scale			x			
Chest tube output			x	x		
UDPB				x		
Additional endpoints						
Ventilation time				x	(x)	(x)
Duration of ICU stay				x	(x)	(x)
Hospital length of stay					x	(x)
All-cause mortality				x	(x)	(x)
AEs and SAEs				x	x	x
Treatment-emergent events				x	x	x
Quality of life	X					x
Unblinding			x		x	

*AEs* Adverse events, *CPB* Cardiopulmonary bypass, *DAOH* days alive and out of hospital, *DC* discharge, *ICU* intensive care unit, *POD* postoperative day, *SAEs* serious adverse events, *UDPB* universal definition of perioperative bleeding

() if needed

^a^Activated clotting time, PT, aPTT, INR, fibrinogen activity via Clauss assay, ROTEM EXTEM CT, ROTEM EXTEM MCF, ROTEM FIBTEM MCF, thrombin generation

^b^Only for first platelet administration

### Setting

The pilot will take place at two Canadian academic tertiary care centers with major cardiac surgical programs, Toronto General Hospital (Toronto, Ontario) and Kingston Health Sciences Centre (Kingston, Ontario).

### Eligibility criteria

Adult (≥ 18 years old) patients undergoing elective cardiac surgery with CPB will be eligible for inclusion if they are planning to undergo at least moderately complex surgery *or* have a preoperative platelet count ≤ 150,000 × 10^6^/L (this is a group at high risk of requiring platelet transfusions post-CPB) [2]. Moderately complex index surgery is defined as (i) repair/replacement of more than one valve; (ii) aorta (root/ascending/arch) replacement; (iii) any combination of coronary artery bypass grafting, valve repair/replacement, or aorta (root/ascending/arch) replacement; or (iv) re-do procedures consisting of a repair or revision of a prior cardiac intervention.

Patients will be excluded if DCSP are not going to be available at the time of surgery or if the patient (i) has a congenital or acquired hemostatic disorder (including platelet refractoriness due to anti-platelet and anti-human leukocyte antigen [HLA] antibodies) and/or requires specially matched platelets (including patients with anaphylaxis to blood due to Immunoglobulin A [IgA] deficiency with anti-IgA antibodies), (ii) has known contraindications to heparin, thereby excluding cases where non-reversible anticoagulants (i.e., argatroban) are used, (iii) is on warfarin or direct oral anticoagulants (dabigatran, rivaroxaban, apixaban or edoxaban) within 3 days prior to surgery, (iv) is on antiplatelet drugs within 5 days prior to surgery (excluding acetylsalicylic acid [ASA]), (v) refuses allogeneic blood products, (vi) has a known pregnancy, or (vii) is enrolled in another interventional clinical trial where routine care and management are altered, (vii) is hemodynamically unstable defined as critical care admission or vasopressor or inotrope dependence prior to index surgery, (viii) has a pre-operative requirement for, or expected post-operative dependence upon, mechanical circulatory support (i.e., intra-aortic balloon pump, ventricular assist device).

### Recruitment

During the surgical preadmission visit, a member of the patient’s circle of care (e.g., surgeon, anesthesiologist, nurse) will invite all elective cardiac surgical patients meeting eligibility criteria to speak with a research coordinator about potential participation in the study. Interested patients will meet with a research coordinator who will provide study information and, where appropriate, obtain written informed consent prior to the scheduled surgical procedure. Thereafter, the research coordinator will contact the hospital blood bank to ensure the availability of adequate supplies of both types of platelets for each patient on the day of surgery (as randomization will only occur on the day of surgery but ABO-identical cold-stored platelets will be prepared a minimum of 24 h prior to the scheduled surgery—see “Randomization/assignment of interventions” section below).

### Randomization/assignment of interventions

Recruited patients will only be randomized on the day of surgery if the clinical team places an order with the blood bank for platelet transfusion to treat active bleeding during the first 6 h after termination of CPB, and both platelet types are available (Fig. 1). When the platelet order is received, the blood bank technologist or assigned research coordinator will confirm the patient has consented to the study and is enrolled in the trial. The patient will then be randomized and the assigned product will be released [18, 24].

Patients will be randomly assigned (1:1 ratio) to study groups on the day of surgery using a permuted block, stratified (by center) allocation scheme prepared by a biostatistician not involved in the trial. Sequentially numbered sealed envelopes based on the randomization lists will be provided to the blood banks that will be providing the platelet product. This process, which has been successfully used in previous similar studies [25–28], has the advantage of allowing the technologists or coordinators to randomize patients as soon as the order is received without delaying therapy. The spot in the randomization list will not be reused if the patient assigned to it does not receive the platelet product. Once the order for the first platelet product is received and eligibility has been confirmed, the blood bank technologist will open the randomization envelope, and prepare and release the assigned platelet product in tamper-proof, temperature-stable containers that have been validated for this purpose and will be identical for each study arm. To maintain blinding, thermal gloves will be released by the blood bank for product handling and the product label (containing collection and expiration dates and type of platelet) will be obscured.

### Interventions

Patients randomized to the room temperature platelet (RTP) group will receive ABO-identical, pathogen-reduced via INTERCEPT [29], buffy-coat platelets in platelet additive solution maintained at 22 °C for up to 7 days, as per the current standard of care.

Patients randomized to the delayed cold-stored platelet (DCSP) group will receive up to two doses of ABO-identical, pathogen reduced via INTERCEPT, buffy-coat platelets in platelet additive solution maintained at 22 °C for up to 4 days (with agitation) then placed in cold-storage (at 1–6 °C, without agitation) for a minimum of 24 h, with expiration at 14 days after collection.

The duration of treatment is from the initiation of the first platelet transfusion up to 24 h after CPB or receipt of two doses of the product, whichever occurs first. There will be no other changes to participants’ pre-and post-operative management.

The clinical team will then administer the platelets only if the following criteria are met: [23, 30] (a) bleeding is severe enough to necessitate treatment (Bleeding Severity Scale Moderate or higher [31] or chest tube output ≥ 200 mL/h for any 1 h or ≥ 2 mL/kg/h for 2 consecutive hours in the first 6 postoperative hours [32]; (b) heparin is adequately reversed (confirmed by the return of post-protamine activated clotting time [ACT] to within 15% of baseline ACT); and (c) presence of thrombocytopenia (total platelet count < 100,000 × 10^6^) or platelet dysfunction (functioning platelet count < 75,000 × 10^6^ in response to collagen activation, as measured by the point-of-care platelet function assay PlateletWorks currently in use at both sites). Patients can also be treated irrespective of the platelet count or function if bleeding is deemed to be life-threatening, in accordance with institutional coagulation management algorithms for massive bleeding previously validated by our group [23]. Once the decision is made to administer platelets, the administering clinician will put on the insulated gloves provided, retrieve the product from its sealed temperature-controlled container, carry out routine checks, and administer according to usual care including in-line filtration tubing (170–260 µm) primed with normal saline.

If a second platelet order is received during the first 24 h after the termination of CPB, the blood bank will prepare and release the correct product (according to group allocation) as per the first unit (in a tamper-proof container along with insulated gloves). To minimize the theoretical thromboembolic risks of cold-stored platelets, if patients require more than two doses of platelets (expected to occur in < 3% of patients, based on data from a recent trial by our group [23]) or require therapy after the 24-h treatment period has elapsed, the blood bank will prepare and release room temperature platelets for both groups. Additional doses of platelets can be administered if there is at least moderate bleeding [31] and other coagulation abnormalities have been addressed as per the standardized coagulation management protocol [23].

### Blinding

This will be a blinded randomized trial, with clinical personnel, patients, family members, and outcome assessors blinded to group allocation. Given that the platelets will be different temperatures (but identical in appearance), an unblinded blood bank technologist, who will not have access to the collected study data, will blind the platelets by placing them in an insulated tamper-proof container accompanied by a pair of disposable insulated gloves. Labels on the product indicating different product codes or revealing an extended date of expiration which may result in unblinding will be concealed. The clinician(s) directly handling the product at the time of transfusion will wear insulated gloves to prevent unblinding (simulations have shown this to be an effective strategy). To maintain blinding, the specific type of product administered will not be recorded in the chart. If any of the product doses are returned to the blood bank, the reason for the return and the status of the seal on the tamper-sealed container will be recorded by the technologist. Outcomes are objective and adjudicators will all be blinded, with an independent adjudication committee used for the main trial. Unblinding is permissible when septic, thromboembolic, or other SAEs occur. Under these circumstances, the Principal Investigator will unblind the patient and the clinical team by contacting the unblinded blood bank technician to reveal a patient’s allocated intervention. All episodes of inadvertent unblinding by the clinical team will be recorded as deviations.

### Outcomes

#### Primary feasibility endpoints

1. Adequate recruitment (defined as ≥ 15% of eligible patients enrolled per center per month to target consistent levels of enrollment) [33]
2. Adequate cold-stored platelet supply (defined as appropriate product available at the time of surgery for ≥ 90% of patients randomized to the delayed cold-stored platelet group).
3. Adequate clinician adherence to randomization assignment (defined as > 90% of all patients being administered the assigned product).

Recruitment and protocol adherence are key common progression criteria for internal pilots of efficient RCTs and ensuring adequate supply of cold-stored platelets is a critical metric for ensuring feasibility of the definitive trial. If all primary feasibility endpoints are met, we will proceed with the larger trial. If 1–2 of 3 feasibility endpoints are met, we will decide if the trial is feasible with modifications to the protocol addressing the specific deficiencies in trial design identified in the pilot. The trial will be considered infeasible if all primary feasibility endpoints fall short of their targets and can not be addressed with modifications to the study protocol. We will hold an investigator meeting to examine the type and number of major deviations, and to define protocol changes required based on the feasibility outcomes.

#### Secondary patient-centered outcomes

(i) mean number of allogeneic blood components (ABCs) transfused (including red blood cells, plasma, and platelets) within 24 h of CPB end, with each platelet recorded as 3.5 units based on donor exposure (as 2 buffy coat platelets prepared from 7 donors), (ii) mean number of ABCs within 7 days of CPB, (iii) total number of coagulation factor products administered (fibrinogen concentrate, prothrombin complex concentrates, and activated recombinant factor VII) within 24 h and 7 days of CPB, (iv) incidence of severe to massive bleeding (using a modification of the universal definition of perioperative bleeding [UDPB] in cardiac surgery, and its individual components [34, 35]) within 24 h of CPB, (v) chest tube drainage at 12- and 24-h after chest closure, (vi) total platelet count with a functional platelet count (PlateletWorks) at preoperative baseline, at the time of rewarming on CPB, immediately after CPB at protamine reversal, within 60 min before first product administration, and within 60 min after first product completion, and (vii) changes in international normalized ratio (INR), prothrombin time (PT), activated partial thromboplastic time (aPTT), fibrinogen activity, rotational thromboelastometry (ROTEM) EXTEM clotting time (CT) and maximum clot firmness (MCF), ROTEM FIBTEM MCF and thrombin generation, from within 60 min before to within 60 min after the initiation of the first product administration, (viii) EQ-5D-5L version of EQ-5D™ questionnaire [36] was collected at baseline and at postoperative day (POD) -30, and (ix) case-costing data for each patient.

Additional safety outcomes include (i) incidence of serious treatment-emergent adverse events (AE) from the start of surgery to POD-30, (ii) incidence of thromboembolic events from randomization to POD-30, (iii) duration of ventilation and ventilator-free days up to POD-30, (iv) duration of intensive care unit (ICU) stay up to POD-30, (v) duration of hospitalization up to POD-30, (vi) incidence of death up to POD-30, and (vii) days alive and out of hospital at POD-30.

### Outcome assessment

See Table 1 for a detailed schedule of assessments. The local research assistant will telephone all patients on POD-30 (± 3 days) to collect responses to the EQ-5D-5L tool. All other outcomes will be collected from the patient’s electronic medical record by blinded members of the study/research team.

### Statistical considerations

The pilot trial will randomize 50 adult (≥ 18 years old) patients undergoing at least moderately complex cardiac surgery with CPB and requiring platelet transfusion. Because the decision to administer platelets occurs intraoperatively but informed consent must be obtained pre-operatively, we will need to consent and enroll a larger sample of approximately 150 eligible patients, as we expect 30–40% of enrolled patients to receive platelets based on historical institutional transfusion practices [23]. This is also the ratio we have observed in similarly designed ongoing RCTs being conducted in this patient population (NCT05523297).

### Determination of sample size

This pilot study will include 50 randomized patients, which, accounting for patients who are randomized but not treated and patients who withdraw in both arms, is expected to provide at least 44 evaluable patients, with 22 in each arm. This sample size of 50 patients allows for sufficiently precise estimates of feasibility outcomes [37] in a reasonable timeline. If fewer than 10% (< 5 patients) receive the incorrect product or receive no product, the upper one-sided 95% confidence limit for this percentage will be ≤ 17.3%. In the 25 patients assigned to cold-stored platelets, if at least 90% have product available, the lower confidence limit will be ≥ 77.7%.

### Statistical analysis

For the statistical analysis of the efficacy parameters, the following analysis sets will be considered:

The intention to treat (ITT) population: all randomized patients who receive at least a whole or parts of an IMP dose, irrespective of the number of IMP doses received. We anticipate that ≥ 90% of randomized patients will receive the first dose of IMP. Should a patient receive IMP that is not in concordance with the randomization schedule, the treatment group will be defined according to the randomization (rather than the actual treatment received). The primary outcome and mortality for non-consenting patients will be collected from as many sites as possible, as permitted by the local REB.

The per-protocol (PP) population: all patients in the ITT sample, excluding patients with major protocol deviations. Patients meeting any of the following criteria will be excluded.

- Patients who do not receive at least 80% of the first platelet dose to which they were randomized
- Patients receiving treatment not in concordance with the randomization schedule
- Patients who significantly violate inclusion/exclusion criteria (e.g., did not have cardiac surgery with CPB, randomized after POD-3, etc.)

The restricted safety population (safety analysis set): all patients who receive at least one whole or parts of the first platelet dose (i.e., the ITT population).

A final decision about the classification of protocol deviations as major and minor and their consequences regarding the assignment of patients to analysis populations will be made during the blinded data review meeting prior to unblinding for the final analyses by the lead PI.

The ITT analysis sample is considered the primary sample for analysis of the primary endpoint. The evaluation of the primary endpoint will additionally be performed for the PP sample.

The data will be analyzed using protocols established for safeguarding privacy and confidentiality. These include password-protected computers, locked file cabinets for hardcopies, and no public discussion of individual cases. There will be no linkages to other datasets, and no disclosures of person identifiers. Reports will present aggregate statistics only.

In the pilot study, we will estimate rates and percentages for feasibility outcomes, along with lower one-sided 95% CIs where high percentages are desirable (e.g., enrollment rate) and upper one-sided CIs where low percentages are desirable (e.g., loss to follow-up, protocol deviations). Point estimates will be compared to our predetermined targets to make decisions on feasibility and the need for changes to the protocol.

Although this study will make no formal inferences on the definitive trial's primary and secondary outcomes, we will present outcomes in tables and graphs (e.g., a histogram for DAOH-30). The differences in means or percentages between the intervention and control groups will be estimated, along with a parametric or bootstrapped (for DAOH) 95% CIs.

The feasibility outcomes of the pilot study will inform the conduct of the definitive trial. Specifically, if all 3 of our primary feasibility outcomes are achieved, we will proceed to a larger trial with minimal or no protocol modifications. If only a portion of our primary feasibility outcomes are achieved, we will hold an investigator meeting and ascertain what aspects of the protocol should be modified, and if a second pilot trial is required in the face of significant protocol modifications prior to proceeding with a larger trial. If none of our primary feasibility outcomes are achieved, we are unlikely to be able to proceed with a larger trial [33, 38]. If we proceed to the definitive trial, all outcome data derived from the pilot study pertaining to the definitive trial’s primary, secondary, and safety outcomes will contribute to the definitive trial's dataset.

No interim analysis will be conducted. We will assess enrollment, randomization, and treatment rates by sex and institution but, because of the small sample size, analyses of the pilot study will not examine subgroups. In the main study, heterogeneity of treatment effect will be assessed by examining possible interaction effects by sex (male/female) and surgical complexity (simple/complex surgery).

### Data management and auditing

The Anesthesia Clinical Trials Unit at Toronto General Hospital will serve as the study coordinating center. Source records will be preserved for the maximum period of time required by local regulations. Authorized study staff (e.g., sub-investigators, clinical research coordinators/assistants, nurses) will enter study data into an electronic case report form (eCRF) for each patient enrolled as documented in the Delegation of Authority Log. A validated institutional REDCap interface will be used. Monitors will perform source data verification (SDV). Discrepancies and queries can only be corrected by the Investigator(s) or other authorized site personnel. Manual checks will be performed and programs run throughout the study until the data is clean and the database is ready for lock. All discrepancies will be resolved prior to database lock. There will be a final run of the programmed checks to ensure all discrepancies are closed out, SDV will be confirmed as complete by the monitor, and all eCRFs will be approved by the Investigator prior to database lock.

### Monitoring and auditing

We will employ an Independent Data Safety Monitoring Committee (IDSMC) tasked with ensuring participant safety. The IDSMC will be independent of the study sponsor and competing interests. If any study findings demonstrate concerns regarding platelet quality, efficacy, or safety, an investigator meeting will be held to review the next steps, which may include an additional meeting of the IDSMC. The Investigator will make all study-related source data and records available to a qualified quality assurance auditor or REB and regulatory inspectors, after reasonable notice.

### Harms

The condition of the patient will be monitored throughout the study. At each visit, whether scheduled or unscheduled, Adverse Events (AEs) will be elicited using a standard non-leading question. In addition, the Investigator will check the patient records for any documented event. Any AE that occurs during the study will be noted in detail on the appropriate pages of the CRF. The Investigator will grade the severity of all AEs (mild, moderate, or severe), the seriousness (non-serious or serious), and the likelihood that they were related to the administered product (causality). The investigator will also assess the expectedness of each (expected or unexpected). The Investigator will provide detailed information about any abnormalities and about the nature of and reasons for any action taken as well as any other observations or comments that may be useful for the interpretation and understanding of an AE. Importantly, for any AE thought to be related to platelet transfusion, local policies for notifying institutional Blood Banks, Canadian Blood Services, the Transfusion Transmitted Injuries Surveillance System, and the Public Health Agency of Canada will be followed. Transfusion reactions will be classified and graded by the International Society of Blood Transfusion haemovigilance criteria (Standardized definitions | The International Society of Blood Transfusion (ISBT) (isbtweb.org)).

### Ethics and dissemination

This study will be conducted in accordance with the ethical principles laid down in the Declaration of Helsinki. The study protocol and any subsequent amendment(s) will be submitted to an REB and to Health Canada. Following approval by Health Canada and the hospital Research Ethics boards, patients will be fully notified about potential risks as part of the informed consent process well before surgery. Eligible patients will be approached by a research coordinator in surgical preadmission clinics for written informed consent. The Investigator will ensure that the patient’s confidentiality is preserved. On CRFs or any other documents submitted to the Sponsor, the patients will not be identified by their names, but by a unique patient identifier. Documents not intended for submission to the Sponsor (e.g., the confidential patient identification code list, original consent forms, and source records) will be maintained by the Investigator in strict confidence. Only the Investigator and statistical team will have access to the final trial dataset and in the event of safety concerns the members of the IDSMC. The results of this study will be published and may be presented at scientific meetings. Authors meeting the International Committee of Medical Journal Editors Guidelines for authorship will be included in relevant publications. Due to the small sample size and potential patient identifiers in the dataset, no additional access to patient-level data will be granted outside of the study team. Any protocol amendments will be communicated to the REB, IDSMC, trial registries, and individual participants.

### Trial status

The trial received a No Objection Letter from Health Canada on February 8, 2023, and REB approval via Clinical Trial Ontario on May 18, 2023. The trial is registered at ClinicalTrials.gov (NCT 06147531). Trial recruitment will begin in 2024 and is expected to be completed by 2026.

## Discussion

Cold-stored platelets have been extensively studied in preclinical settings, and pilot trials in humans suggest at least comparable efficacy with no increased risk for adverse outcomes compared to room-temperature platelets [9, 18, 24, 39]. However, the safety and efficacy of cold-stored platelets have not been evaluated in large clinical trials and these products are not currently approved for use in Canada. Although they may have hemostatic superiority over conventional room-temperature platelets, there is a possibility that cold-stored platelets may increase the risk of thromboembolic events (as they are hemostatically more activated upon transfusion). However, this risk is largely theoretical and has not been demonstrated in prior studies [18, 40]. Nevertheless, we have taken several steps to minimize this risk and to identify it early should it occur. Additionally, while cohort studies have been published examining the impact on patient care of introducing delayed cold-stored platelets during times of shortages, large studies systematically measuring efficacy and safety are lacking [18].

The co-primary feasibility endpoints are adequate enrollment, adequate cold-stored platelet supply, and adequate clinician adherence to randomization assignment. We selected these outcomes because they will indicate what, if any, are the most important protocol modifications required before undertaking a large, definitive trial. Specifically, recruitment and protocol adherence are key common progression criteria for internal pilots of efficient RCTs [33], and confirming an adequate supply of cold-stored platelets at the time of transfusion is a critical metric for ensuring the feasibility of the definitive trial.

The planned primary outcome for the definitive study will be the mean number of ABCs transfused (including red cells, plasma, and platelets) within 24 h of CPB end. This outcome is an accepted measure of hemostatic efficacy that captures clinically relevant differences between study arms. It has been used as a primary outcome in a large randomized controlled trial comparing fibrinogen concentrate to cryoprecipitate in cardiac surgical patients in Canada [26, 28]. This outcome was used to support Canadian regulatory approval of fibrinogen concentrate in cardiac surgical patients, demonstrating its validity and importance for key regulatory stakeholders, and was a recommended outcome from the recent NHLBI Hemostasis Clinical Trial Outcomes Working Group [41].

Patient management in the control group will reflect current practice and patient management in the intervention group will reflect how delayed cold-stored platelets will be used in practice. For these reasons, the definitive study will have good external validity.

Blood suppliers have indicated an urgent need for innovative platelet storage modalities to extend platelet lifespan without compromising efficacy or safety, a problem delayed cold-stored platelets may be able to address [24, 42]. A well-designed, multicentre RCT is required to assess the comparative efficacy and safety of cold-stored and room-temperature buffy-coat prepared platelets. If the results of the definitive RCT are positive, they will be used to support future licensing by CBS, Canada’s main blood supplier of this product in Canada. Moreover, because buffy-coat prepared platelets are the “industry standard”, findings of such a trial will also be applicable to most other countries, including the UK, Europe, Australia, and most of South America. Buffy coat, and pooled, pathogen-reduced platelets by INTERCEPT are the most common platelet products currently utilized worldwide. If this trial has a positive or non-inferior result, it may result in platelets being able to be stored near operating rooms in controlled fridges for near-patient access, and due to their longer shelf life, the feasibility of stocking smaller hospitals without platelet shakers and with lower transfusion volumes may be increased. The proposed pilot study is designed to determine optimal trial procedures, including the provision of delayed cold-stored platelets at participating hospitals, for the definitive trial.

## Data Availability

Requests for the full study protocol or related study information may be made to the corresponding study author. No individual patient-level data will be shared with external parties. Data sharing is not applicable to this article as no new datasets were generated or analyzed during the current study.

## Abbreviations

ABC: Allogeneic blood components
ACT: Activated clotting time
ABO: Blood group system
ADP: Adenosine diphosphate
ADR: Adverse drug reaction
AE: Adverse event
ALP: Alkaline phosphatase
ALT: Alanine transaminase
aPTT: Activated partial thromboplastin time
ASA: Acetylsalicylic acid
AST: Aspartate aminotransferase
BMI: Body Mass Index
CBC: Complete blood count
CBS: Canadian Blood Services
CI: Confidence interval
COVID-19: Coronavirus disease of 2019
CPB: Cardiopulmonary bypass
CRF: Case Report Form
CRO: Clinical Research Organization
CT: Clotting time
DAOH: Days alive and out of hospital
eCRF: Electronic Case Report Form
EDC: Electronic Data Capture
FDA: Food and Drug Administration
FP: Frozen plasma
FXIII: Factor XIII
GCP: Good Clinical Practice
HLA: Human leukocyte antigen
IB: Investigator’s brochure
ICF: Informed consent form
ICU: Intensive care unit
IDSMC: Independent Data Safety Monitoring Committee
IgA: Immunoglobulin A
IMP: Investigational Medicinal Product
INR: International normalized ratio
ITT: Intention to treat
MedDRA: Medical Dictionary for Regulatory Activities
MCF: Maximum clot firmness
MPV: Mean platelet volume
NAP-2: Neutrophil-activating peptide 2
NHLBI: National Heart, Lung, and Blood Institute
PAI-1: Plasminogen activator inhibitor-1
PBS: Phosphate-buffered saline
PCC: Prothrombin complex concentrates
PFA: Paraformaldehyde
PF-4: Platelet factor 4
PI: Principal investigator
PLT: Platelets
POD: Postoperative day
PP: Per-protocol
PRP: Platelet-rich plasma
PT: Prothrombin time
RBC: Red blood cells
RCT: Randomized controlled trial
REB: Research Ethics Board
rFVIIa: Activated recombinant factor VII
Rh: Rhesus factor
ROTEM: Rotational thromboelastometry
SAE: Serious adverse event
SAF: Safety analysis population
SAP: Statistical analysis plan
SD: Standard deviation
SDM: Substitute decision maker
SDV: Source Data Verification
SMQ: Standardized MedDRA Queries
SUSAR: Suspected Unexpected Serious Adverse Reaction
UDPB: Universal definition of perioperative bleeding
vWF: Von Willebrand Factor
